# Neuroblastoma Invasion Strategies Are Regulated by the Extracellular Matrix

**DOI:** 10.3390/cancers13040736

**Published:** 2021-02-10

**Authors:** Cian Gavin, Nele Geerts, Brenton Cavanagh, Meagan Haynes, C. Patrick Reynolds, Daniela Loessner, Andrew J. Ewald, Olga Piskareva

**Affiliations:** 1 Cancer Bio-Engineering Group, Department of Anatomy and Regenerative Medicine, RCSI University of Medicine and Health Sciences, Dublin D02 YN77, Ireland; ciangavin@rcsi.com (C.G.); nelegeerts@rcsi.com (N.G.); 2 Cellular and Molecular Imaging Core, RCSI University of Medicine and Health Sciences, Dublin D02 YN77, Ireland; brentoncavanagh@rcsi.com; 3 Center for Cell Dynamics, Department of Cell Biology, School of Medicine, Johns Hopkins University, Baltimore, MD 21205, USA; mhayne11@jhmi.edu (M.H.); andrew.ewald@jhmi.edu (A.J.E.); 4 Cancer Center, School of Medicine, Texas Tech University Health Sciences Center, Lubbock, TX 79416, USA; patrick.reynolds@ttuhsc.edu; 5 Departments of Pediatrics and Internal Medicine, School of Medicine, Texas Tech University Health Sciences Center, Lubbock, TX 79416, USA; 6 Departments of Chemical Engineering and Materials Science and Engineering, Faculty of Engineering, Monash University, Melbourne, VIC 3800, Australia; daniela.loessner@monash.edu; 7 Department of Anatomy and Developmental Biology, Faculty of Medicine, Nursing and Health Sciences, Monash University, Melbourne, VIC 3800, Australia; 8 Sidney Kimmel Comprehensive Cancer Center, Cancer Invasion and Metastasis Program, Department of Oncology, School of Medicine, Johns Hopkins University, Baltimore, MD 21205, USA; 9 School of Pharmacy and Biomolecular Sciences, RCSI University of Medicine and Health Sciences, Dublin D02 YN77, Ireland; 10 National Children’s Research Centre, Our Lady’s Children’s Hospital Crumlin, Dublin D12 8MGH, Ireland

**Keywords:** neuroblastoma, organoids, 3D invasion assay, invasion phenotype, extracellular matrix environment (ECM)

## Abstract

**Simple Summary:**

Paediatric cancer research in general and neuroblastoma, in particular, has minimal preclinical models of metastasis. As 50% of primary neuroblastomas have already metastasised at the time of diagnosis, it is important to develop models to understand the molecular mechanisms of neuroblastoma metastasis. Here, we describe a novel patient-derived xenograft (PDX)- and cell line-based organoid model. We found that the extracellular matrix (ECM) composition influenced the growth, viability and local invasion of organoids. PDX-derived neuroblastoma organoids displayed four various invasion phenotypes which were dependent on the local microenvironment, while cell lines were more restricted in their invasion strategies. These data support the use of organoid cultures for studying the biology and molecular basis of neuroblastoma invasion into normal tissues.

**Abstract:**

Neuroblastoma is a paediatric malignancy of the developing sympathetic nervous system. About half of the patients have metastatic disease at the time of diagnosis and a survival rate of less than 50%. Our understanding of the cellular processes promoting neuroblastoma metastases will be facilitated by the development of appropriate experimental models. In this study, we aimed to explore the invasion of neuroblastoma cells and organoids from patient-derived xenografts (PDXs) grown embedded in 3D extracellular matrix (ECM) hydrogels by time-lapse microscopy and quantitative image analysis. We found that the ECM composition influenced the growth, viability and local invasion of organoids. The ECM compositions induced distinct cell behaviours, with Matrigel being the preferred substratum for local organoid invasion. Organoid invasion was cell line- and PDX-dependent. We identified six distinct phenotypes in PDX-derived organoids. In contrast, NB cell lines were more phenotypically restricted in their invasion strategies, as organoids isolated from cell line-derived xenografts displayed a broader range of phenotypes compared to clonal cell line clusters. The addition of FBS and bFGF induced more aggressive cell behaviour and a broader range of phenotypes. In contrast, the repression of the prognostic neuroblastoma marker, *MYCN*, resulted in less aggressive cell behaviour. The combination of PDX organoids, real-time imaging and the novel 3D culture assays developed herein will enable rapid progress in elucidating the molecular mechanisms that control neuroblastoma invasion.

## 1. Introduction

Neuroblastoma (NB) is the most common solid tumour in children [1,2]. At the time of diagnosis, metastatic spread is seen in half of the patients with primary neuroblastomas, which are in a high-risk category. Despite an intensive multimodal treatment regime, 50–60% of high-risk patients will relapse, which usually occurs within two years of diagnosis [3].

The spread of tumour cells, believed to account for up to 90% of cancer-related morbidities [4], is a heterogenous multistep process that is yet to be fully understood. The concept of cancer cell invasion is an adaptive and dynamic process that depends on various structural, molecular and microenvironmental conditions and as such requires adequate experimental models. Our understanding of this process in NB is limited in part due to a lack of appropriate model systems [5].

To date, studies investigating NB cell migration and invasion have relied on the widely used transwell and scratch wound assays that are easy to carry out at low cost. These assays have limitations which lead us to assess their effectiveness for modelling NB invasion and metastasis. First and foremost is that both assays involve the use of cancer cells grown in 2D monolayer cultures. These cultures fail to accurately recapitulate the 3D architecture of solid tumours, where malignant cells are heterogeneously exposed to different biochemical (nutrients, oxygen) and biophysical (stiffness) properties of the microenvironment. Furthermore, 2D cultures result in cellular homogeneity and hence are disadvantageous in modelling intratumour heterogeneity [6]. The lack of appropriate model systems to investigate local tumour cell invasion in NB prompted us to use a 3D in vitro invasion assay using patient-derived materials.

Subpopulations of malignant cells within a tumour display different phenotypic features, including cell morphology, motility, gene expression, drug resistance and metastatic potential [7]. This is known as intratumour heterogeneity, a hallmark of all malignancies, including NB, and it is essential that preclinical disease models maintain this cellular heterogeneity. In the past, the most widely used models of high-risk NB were cancer cell lines, subcutaneous xenograft tumours derived from these cell lines, and genetically engineered mouse models, all of which have major limitations in maintaining intratumour heterogeneity. Cell lines are derived through clonal expansion of tumour cells, whereby those that survive in culture have up-regulated pro-survival genes. This results in a homogeneous population of cells and is among the reasons that cell lines often fail to predict the clinical efficacy of targeted anti-cancer therapies used in clinical trials. Despite this, in 2016, 82% of published preclinical studies employed cell line-derived tumour models [5,8]. On the other hand, genetically engineered mouse models are often developed under the control of a specific oncogenic driver (*MYCN*, *ALK*) and hence do not capture the genetic diversity of human NB, which has many oncogenic drivers, including copy number alterations, chromosomal imbalances and *TERT* rearrangements [9]. These limitations are partially overcome by using patient-derived xenograft (PDX) models, which are created by the direct transplantation of human tumour material into immunodeficient mice and maintained by in vivo passaging [10]. Unlike the aforementioned approaches, PDXs maintain the histopathological features, genetic/epigenetic characteristics and anti-cancer drug sensitivities of their parental tumours [11,12,13,14,15,16]. Thus, PDXs, as a model system, maintain interpatient and intratumour heterogeneity. Because patient-derived NB tissue is scarce, PDXs provide a particularly relevant source of native tumour tissue for biological studies.

Here, we report a 3D in vitro model of local tumour invasion in NB. We maximise the experimental yield of PDXs by isolating tumour organoids and growing them in hydrogel-based 3D models that mimic the extracellular matrix (ECM). We show that the ECM composition modifies the growth, migration, viability and local invasion of NB organoids by performing real-time 3D culture assays. Lastly, we benchmark NB organoids isolated from PDXs against clonal expansion of cell lines and cell line-derived xenografts and investigate the role of tumour-biological factors (soluble factors, *MYCN*).

## 2. Results

### 2.1. Cell Invasion Strategies of NB Organoids in ECM-Mimicking Matrices

**Figure 1:**
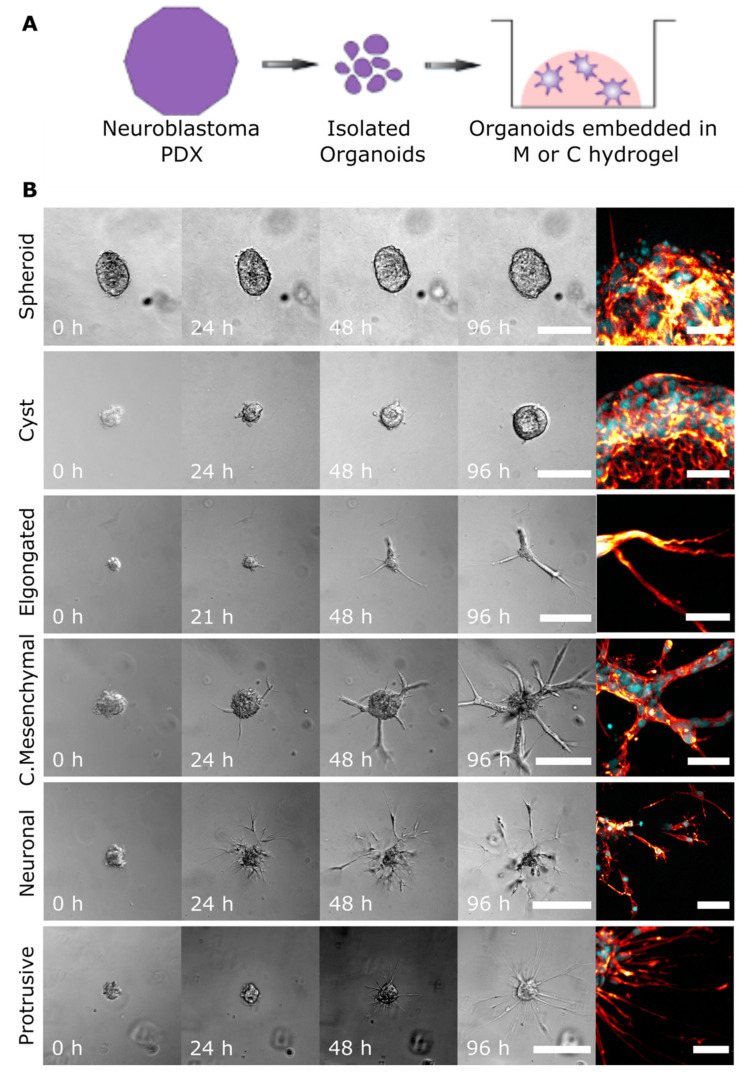
Neuroblastoma (NB) organoids are heterogenous and display a spectrum of distinct invasion strategies in 3D assays. (**A**) Schematic of isolation and 3D culture of human neuroblastoma clusters. Matrigel, M, Collagen, C hydrogels. (**B**) Representative DIC time-lapse and confocal images of organoids isolated from Felix-patient-derived xenograft (PDX) and grown in 3D extracellular matrix (ECM) cultures. Confocal images, taken on day 5 of 3D culture, show nuclei stained with DAPI (cyan) and actin filaments stained with phalloidin (red). Based on the morphology NB, organoids were classified as non-invasive (spheroid, cyst) or invasive (elongated, collective mesenchymal, neuronal or protrusive). For spheroid and cyst: DIC scale bars = 100 μM, confocal scale bars = 30 μM, elongated DIC scale bars = 150 μM, confocal scale bars = 30 μM. For all remaining DIC images: scale bars = 200 μM, confocal scale bars = 50 μM.

To investigate the effect of the local ECM microenvironment on the invasive behaviour of organoids derived from human PDXs, we performed time-lapse microscopy of NB organoids embedded in 3D ECM cultures over 96 h (Figure 1A). We observed that NB cells displayed a spectrum of distinct behaviours within the organoid populations; representative images of each are shown in Figure 1B. To classify the observed phenotypes, we used previously published terminology that is based on the visual appearance of organoids and the presence and cellular distribution of actin filaments [17,18,19,20,21,22,23,24]. NB organoids commonly invaded as collective strands where adhesion between neighbouring cells was maintained. Confocal imaging confirmed the presence of one or several actin-rich protrusions indicative of cancer cells with mesenchymal traits. Thus, we termed this cellular strategy of invasion “collective mesenchymal”. We also observed collective strand invasion in “elongated” organoids, when no multicellular branching was documented but cells within these organoids migrated in a common direction with a leading multicellular stream. Therefore, this phenotype may be molecularly distinct from the collective mesenchymal phenotype. In comparison, collective mesenchymal organoids extended their multicellular strands in multiple directions, and these strands commonly branched (Figure 1B).

We identified organoids with dispersed cells extending neurite-like processes into the ECM and termed this phenotype “neuronal”. This cellular strategy of invasion was morphologically comparable to that seen in glioma, where cells invade as a collective network with transient cell–cell interactions both in 3D in vitro and in vivo models [22,23]. We also identified organoids that maintained a round central cell mass while extending long thin protrusions into the ECM and termed this phenotype “protrusive”. The radial protrusions observed were actin-rich and often without nuclei, which may be unique to NB organoids (Figure 1B). When nuclei were seen within these protrusions, they were individually migrating or loosely followed by multicellular streams.

In addition to the invasive phenotypes described above, phenotypic heterogeneity was also seen in non-invasive organoids. Organoids that maintained a round cellular mass were termed “spheroid”, while round organoids that developed a lumen were termed “cyst”. In a similar experimental setup, cyst formation with a bi-layered epithelial structure was reported as a model of epithelial duct formation [25]. Although we did not investigate whether the cyst-like structures were bi-layered, we speculate, given the origin of NB, that they may in fact be neuroepithelial in nature. Neuroepithelial cyst formation in hydrogels has been described as a model of neural tube development in vivo [26]. Considering the multipotency of the cells of origin for NB, the distinct invasive behaviours observed in organoids isolated from PDXs suggests that the heterogeneity of NB is retained in our 3D approach.

### 2.2. Invasion of NB Organoids Depends on the PDX of Origin and ECM Composition

To further elucidate the invasive behaviour of NB organoids, we isolated NB cells from four different PDXs, namely, 424x, 573x, 603x and Felix (Table 1, File S1) and embedded them into two different matrices (Matrigel, collagen type-I) for time-lapse microscopy analysis. Matrigel is primarily composed of laminin (~60%), collagen IV (~30%), nidogen (~5%), heparan sulphate proteoglycan (perlecan, ~3%) and entactin (~1%) and contains undefined amounts of growth factors, including bFGF [27]. We used growth factor-reduced Matrigel to limit the impact of growth factors on cell invasion.

**Figure 2:**
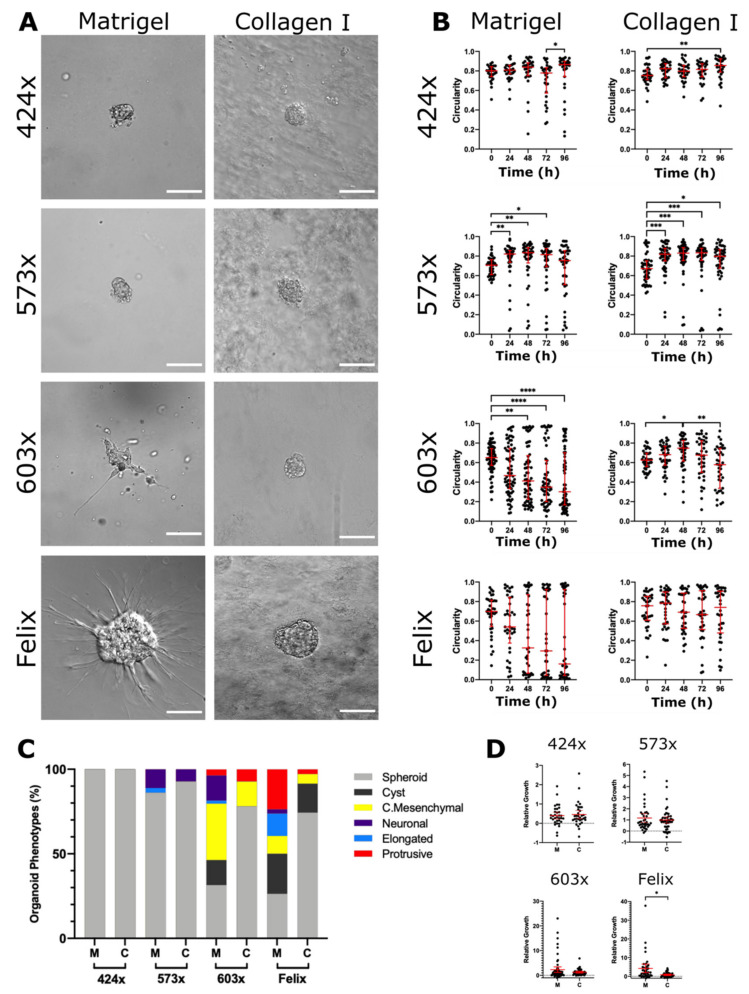
ECM microenvironments modulate the pattern of collective invasion and local dissemination in neuroblastoma cells. (**A**) Representative DIC images of organoids isolated from neuroblastoma PDXs and grown in 3D ECM cultures at 96 h. Based on morphology NB, organoids were classified as non-invasive (spheroid, cyst) or invasive (elongated, collective mesenchymal, neuronal or protrusive). For spheroid and cyst: DIC scale bars = 100 μM; elongated scale bars = 150 μM DIC. For other DIC images: scale bars = 200 μM. (**B**) Organoid circularity was measured at 96 h in Matrigel and collagen (Kruskal–Wallis and Dunn’s post-hoc tests; horizontal bars represent median and interquartile range). (**C**) Bar charts represent phenotype classifications. (**D**) The relative growth of organoids was calculated by dividing the difference in area between 0 h and 96 h by the area at 0 h. Each dot represents the relative growth of one organoid (Matrigel: n = 32 for 424x, *n* = 41 for 573x, *n* = 3 for 603x, *n* = 38 for Felix; collagen: *n* = 35 for 424x, *n* = 46 for 573x, *n* = 40 for 603x, *n* = 36 for Felix). Error bars indicate the mean ± 95% confidence interval. Asterisks indicate statistical significance obtained using Brown–Forsythe ANOVA with T3 Dunnett post-hoc test (ns, not significant, * *p* < 0.05, ** *p* < 0.01, *** *p* < 0.001, **** *p* < 0.0001). Scale bars = 100 μM.

The extent of invasion in both 3D ECM cultures was PDX-dependent. 424x organoids exclusively formed spheroids in both matrices (Figure 2A) and hence were non-invasive. To confirm our microscopic observations, we measured the circularity of 424x organoids and found high circularity values (Figure 2B) and a predominant spheroid phenotype (Figure 2C). We identified a small number of 424x organoids with transient cellular protrusions, reflected by lower circularity values (Figure S1.2 in File S1).

573x organoids also predominantly formed non-invasive spheroids in both Matrigel (86%) and collagen (93%) matrices (Figure 2A). However, a small proportion of 573x organoids invaded both matrices and were classified as neuronal due to the presence of neurite-like processes (Figure S1.3 in File S1). 573x organoids began to invade within 24 h. We also found one elongated organoid in Matrigel. Plotting the circularity of 573x organoids over time supported our microscopic observations. The vast majority formed spheroids rapidly, resulting in a significantly increased circularity after 24 h in both matrices (Figure 2B). As the smaller proportion of invasive organoids continued to lose their circularity, the degree of significance declined over time in both matrices; however, Matrigel contained more invasive organoids (14%) compared to collagen (7%) (Figure 2C). The exposure to different ECM compositions did not induce distinct phenotypes in 573x organoids.

In contrast, 603x organoids displayed an ECM-dependent invasion that was phenotypically heterogeneous (Figure 2A). 603x organoids preferentially invaded Matrigel, with 56% of organoids classified as invasive, compared to collagen, in which only 22% of organoids were invasive. This preference was confirmed by their circularity, with most 603x organoids rapidly losing their circularity in Matrigel, significantly after 48 h (Figure 2B). In collagen, organoid circularity increased over 48 h, followed by a significant decrease up to 96 h. We found a greater variety of organoid phenotypes in Matrigel, where all six morphological phenotypes were identified, compared to collagen, with only four phenotypes (Figure 2C). Although there was no significant difference in the relative growth of 603x organoids between both matrices, the greatest growth occurred in Matrigel (Figure S1.4 in File S1).

Felix organoids also displayed a profound preference for Matrigel when invading their local ECM (Figure 2A), with 50% of organoids being invasive. Their circularity showed a bimodal behaviour in Matrigel, with half of Felix organoids losing their circularity as they invaded their local ECM (Figure 2B). In contrast, the majority of Felix organoids grown in collagen maintained high circularity values, indicative of the predominant non-invasive spheroid (74%) and cyst (17%) phenotypes (Figure 2C). The relative growth of Felix organoids supported Matrigel as a promoter of invasion, with a significantly increased growth compared to collagen (Figure 2D, Figure S1.5 in File S1)

### 2.3. Invasion, Growth and Viability of NB Organoids Depend on Soluble Factors

Next, we explored the impact of serum and growth factors in the microenvironment on the behaviour of organoids. To investigate whether these nutrients were essential for aggressive behaviour, we selected Felix organoids due to their consistently invasive nature. Felix organoids were grown in two different matrices and their medium supplemented with FBS (10%), basic fibroblast growth factor (bFGF/FGF-2; 2.5 nM) or both (FBS/bFGF). We performed time-lapse microscopy, 3D invasion assays and assessed their viability using ethidium homodimer to evaluate organoid responses to the different microenvironmental conditions.

**Figure 3:**
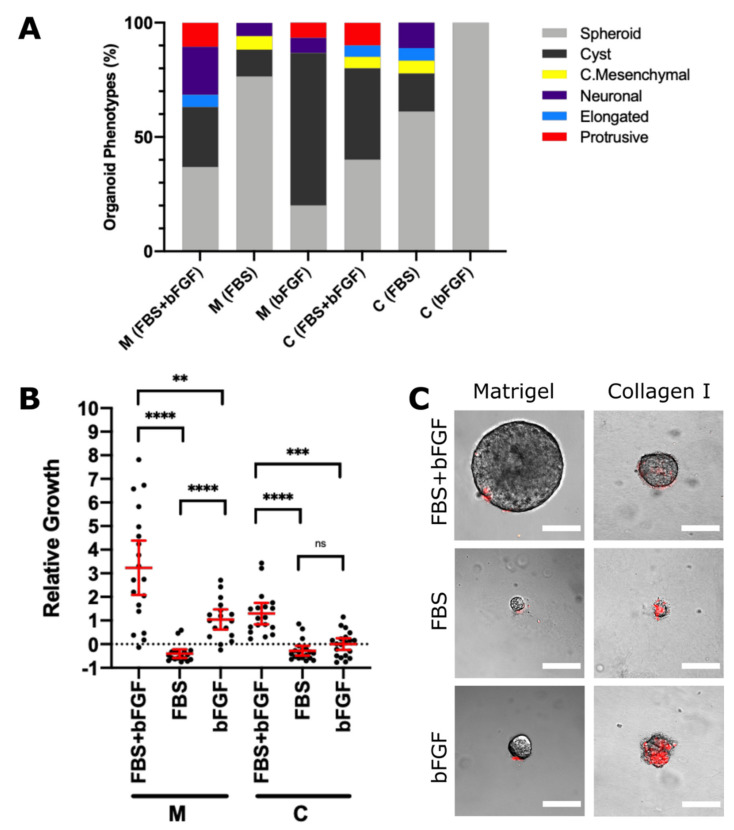
The invasion and growth of NB organoids is dependent on soluble factors in the microenvironment. (**A**) Felix-PDX organoids were cultured in Matrigel or collagen in the presence of FBS + bFGF, FBS or bFGF alone and corresponding bar charts representing phenotype classifications. (**B**) The relative growth of these organoids was calculated by dividing the difference in area between 0 h and 96 h by the area at 0 h. (**C**) Representative images of EHD staining (dead cells: red) of organoids at 120 h. Each dot represents the relative growth of one organoid (Matrigel: *n* = 19 in FBS/bFGF, *n* = 17 in FBS, *n* = 15 in bFGF. Collagen: *n* = 20 in FBS/bFGF, *n* = 17 in FBS, *n* = 14 in bFGF). Error bars indicate the mean ± 95% confidence interval. Asterisks indicate statistical significance obtained using Brown–Forsythe ANOVA with T3 Dunnett post-hoc test (ns, not significant, * *p* < 0.05, ** *p* < 0.01, *** *p* < 0.001, **** *p* < 0.0001). Scale bars = 100 μM.

The presence of FBS and bFGF induced diverse organoid phenotypes in both matrices tested (Figure 3A). In Matrigel, 37% of organoids were classified as invasive in the presence of both FBS/bFGF, while only 12 or 13% of organoids were invasive when FBS or bFGF alone were present, respectively. In contrast, 56% of organoids had an invasive phenotype when both FBS/bFGF were present. Of note, the number of organoids in both matrices was relatively low. In the collagen matrix, invasive organoids were also absent upon bFGF treatment, while invasion was observed with FBS (24% of organoids) and FBS/bFGF treatment (20% of organoids).

The relative growth of Felix organoids showed a similar trend as the phenotype classifications, with organoids grown in both bFGF/FBS revealing the highest growth rate in both matrices (Figure 3B). In Matrigel, bFGF alone significantly increased organoid size compared to FBS alone. Conversely, both bFGF/FBS were required to promote organoid growth in collagen. Most organoids supplemented with FBS decreased in size, suggesting decreased cell viability (Figure 3B, data below dotted line). We confirmed the presence of non-viable cells using ethidium homodimer staining (Figure 3C). The treatment with both bFGF/FBS led to highly viable organoids in our 3D invasion assays. Overall, NB organoid cultures in Matrigel and the simultaneous presence of bFGF and FBS promoted an aggressive behaviour and local cell invasion.

### 2.4. Neuroblastoma Cell Lines Display ECM-Independent Invasion Strategies

**Figure 4:**
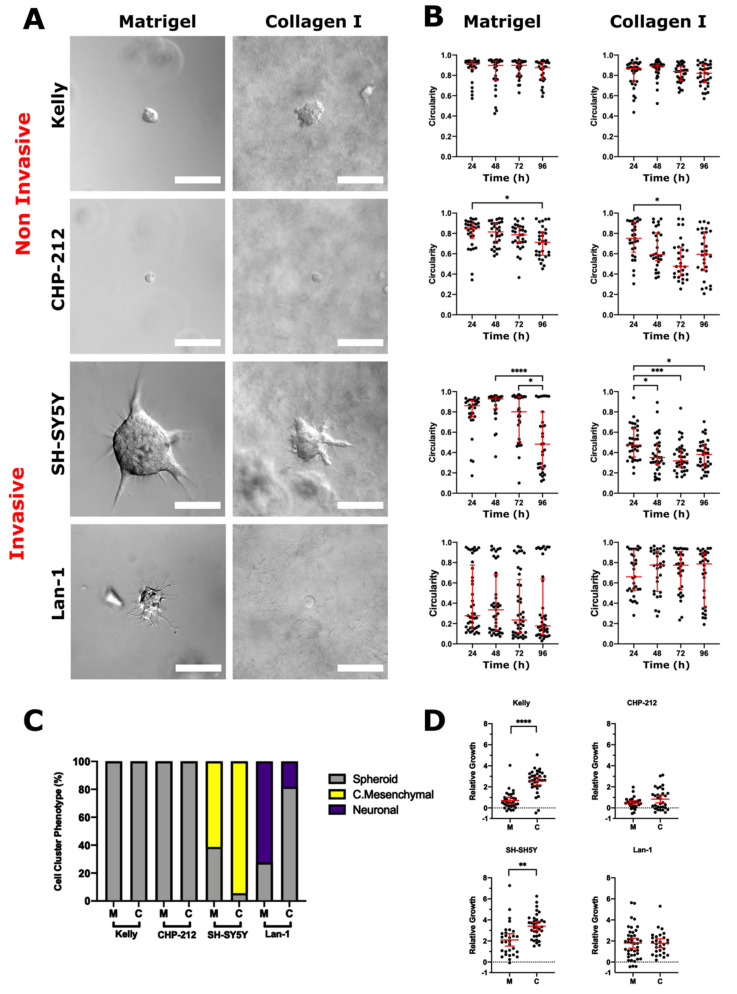
ECM microenvironments modulate the pattern of collective invasion and local dissemination in human neuroblastoma cells. (**A**) Representative DIC images of NB cell clusters grown in 3D ECM cultures at 96 h. Based on the morphology NB cell clusters were classified as non-invasive (spheroid, cyst) or invasive (elongated, collective mesenchymal, neuronal or protrusive). (**B**) Cell cluster circularity was measured at 96 h in Matrigel and collagen (Kruskal–Wallis and Dunn’s post-hoc tests; horizontal bars represent median and interquartile range). (**C**) Corresponding bar charts represent phenotype classifications. (**D**) The relative growth of these clusters was calculated by dividing the difference in area between 0 h and 96 h by the area at 0 h. Each dot represents the relative growth of one cell cluster (Matrigel: *n* = 31 for Kelly, *n* = 30 for CHP-212, *n* = 30 for SH-SY5Y, *n* = 41 for Lan-1; collagen: *n* = 30 for Kelly, *n* = 29 for CHP-212, *n* = 34 for SH-SY5Y, *n* = 34 for Lan-1). Error bars indicate the mean ± 95% confidence interval. Asterisks indicate statistical significance obtained using Brown–Forsythe ANOVA with T3 Dunnett post-hoc test (ns, not significant, * *p* < 0.05, ** *p* < 0.01, *** *p* < 0.001, **** *p* < 0.0001). Scale bars = 30 (Kelly, CHP-212), 50 (Lan-1) and 100 μM (SH-SY5Y).

We next investigated the invasion behaviour of commonly used NB cell lines (*MYCN* amplified (MNA) and non-amplified (nMNA); Table S2.1 in File S2) in Matrigel and collagen (Figure 4). The cell line clusters successfully developed from single cells in both matrices, as determined by time-lapse microscopy. Most cell lines formed clusters of varying sizes, regardless of the matrix (Table 2). We observed only two distinct invasive phenotypes, collective mesenchymal and neuronal. We regarded two of the MNA cell lines, Kelly and CHP212, as non-invasive because they exclusively formed spheroids (Figure 4A). Conversely, Lan1 (MNA) and SH-SY5Y (nMNA) cells had invasive phenotypes.

SH-SY5Y cell clusters were mostly invasive in both matrices (61% in Matrigel and 95% in collagen) (Figure 4C). SH-SY5Y cells exclusively displayed the collective mesenchymal mode of invasion, which was identified by the presence of multicellular branches with protrusive tips.

Lan-1 cell clusters were predominantly classified as invasive neuronal in Matrigel (73%), whereas clusters mostly formed non-invasive spheroids in collagen (82%) (Figure 4C). This cell line behaved similarly to the PDX NB organoids in that regard, showing increased invasion in Matrigel. Our microscopic observations were confirmed by measuring the circularity of clonal cell clusters over time (Figure 4B). The non-invasive cell lines maintained high circularity values, while the invasive cell lines displayed decreased circularity.

Interestingly, the clonal clusters of the NB cell lines did not display the same phenotypic diversity as the PDX organoids in our 3D invasion assays.

### 2.5. Expression Levels of MYCN Are Associated with Aggressive NB Behaviour

**Figure 5:**
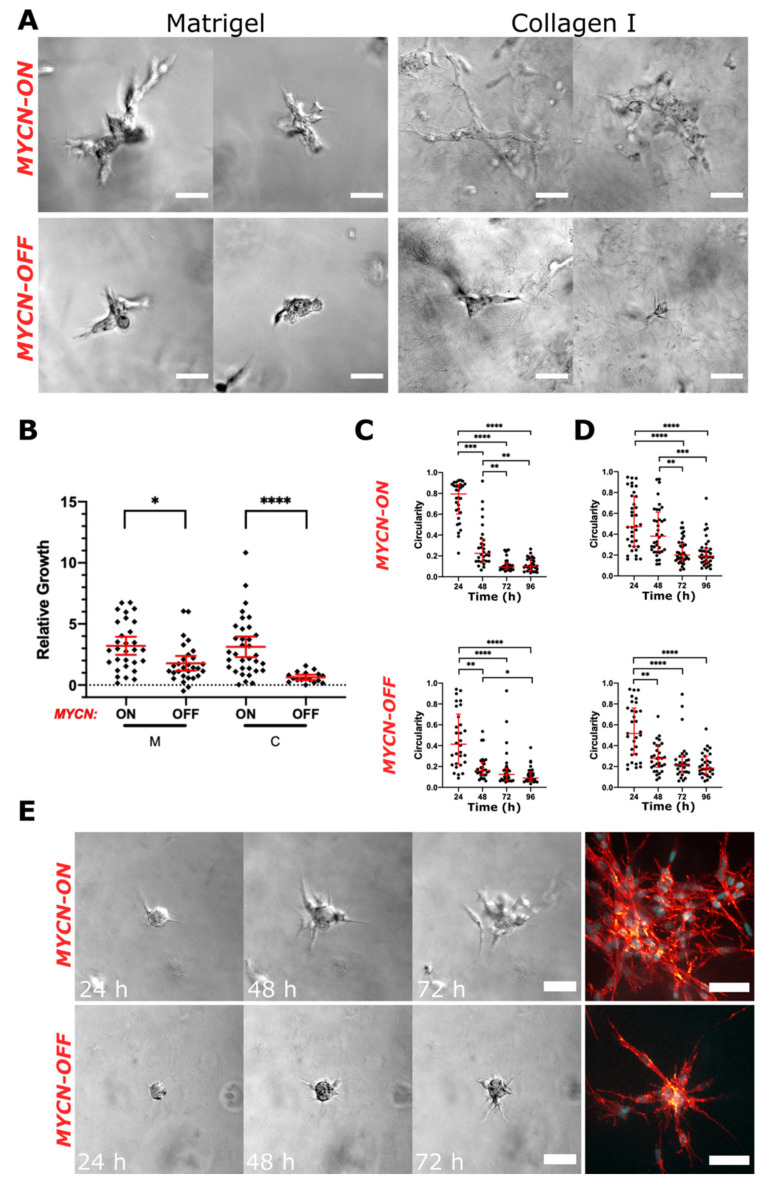
Repressing *MYCN* expression promotes less aggressive cell behaviour in SHEP-Tet21N cells. (**A**) Representative DIC images of untreated SHEP-Tet21N (*MYCN*-ON) and doxycycline-treated (*MYCN*-OFF) cells after 72 h in Matrigel and collagen. (**B**) Relative growth of organoids was calculated by dividing the difference in cluster area at 72 h compared to 24 h, by the area at 24 h (Brown–Forsythe ANOVA with T3 Dunnett post-hoc tests; horizontal bars represent mean ± 95% confidence intervals). Circularity of *MYCN*-ON and *MYCN*-OFF cell clusters was measured at 72 h in Matrigel (**C**) and collagen (**D**) (Kruskal–Wallis and Dunn’s post-hoc tests; horizontal bars represent median and interquartile range). (**E**) Representative DIC time-lapse and confocal micrographs (120 h) comparing SHEP-Tet21N cell clusters with *MYCN*-ON and *MYCN*-OFF; nuclei stained with DAPI (cyan) and F-actin stained with phalloidin (red). In all plots, each dot represents one cell cluster (*MYCN*-ON; *n* = 30 in Matrigel, *n* = 34 in collagen. *MYCN*-OFF; *n* = 29 in Matrigel, *n* = 17 in collagen) and asterisks indicate statistical significance (* *p* < 0.05, ** *p* < 0.01, *** *p* < 0.001, **** *p* < 0.0001). Scale bars = 50 μM.

Genomic amplification of *MYCN* occurs in approximately 20% of NBs and is associated with aggressive disease and poor outcome [31]. Elevated levels of *MYCN* expression may activate genes that drive aggressive cell behaviour. Therefore, we aimed to investigate whether elevated *MYCN* expression promotes the growth and invasion of NB cells. We embedded single SHEP-Tet21N cells that were continuously treated with doxycycline to repress the expression of *MYCN* [32] (*MYCN*-OFF) and untreated cells (*MYCN*-ON) in Matrigel and collagen matrices and determined clonal expansion and cell invasion by time-lapse microscopy over 96 h (Figure 5).

Cell clusters exclusively employed the collective mesenchymal strategy in both matrices (Figure 5A). However, the invasive behaviour appeared more aggressive in *MYCN*-ON cells. Furthermore, *MYCN*-ON cells formed significantly larger clusters compared to *MYCN*-OFF in both matrices (Figure 5B). Measuring the circularity of individual cell clusters confirmed our microscopic observations and illustrated the highly invasive nature of SHEP-Tet21N cells, regardless of the *MYCN* status. The vast majority of cell clusters lost their spherical morphology after 48 h (circularity < 0.4) in Matrigel (Figure 5C) and collagen (Figure 5D). This trend in circularity loss was similar in *MYCN*-ON and *MYCN*-OFF cells; however, the representative time-lapse and confocal micrographs depicted that invasion in fact was more aggressive when *MYCN* transcription is turned “ON” (Figure 5E). These results suggest that cells with high levels of MYCN display an aggressive behaviour in our 3D invasion assay. However, we found that NB organoids isolated from two of the MYCN-amplified PDXs (Figure 2C) did not display an invasive behaviour in our 3D assays, indicating that factors other than MYCN regulate the invasive phenotype.

### 2.6. The In Vivo Tumour Microenvironment Fosters a Distinct Invasion Strategy in NB Cells

**Figure 6:**
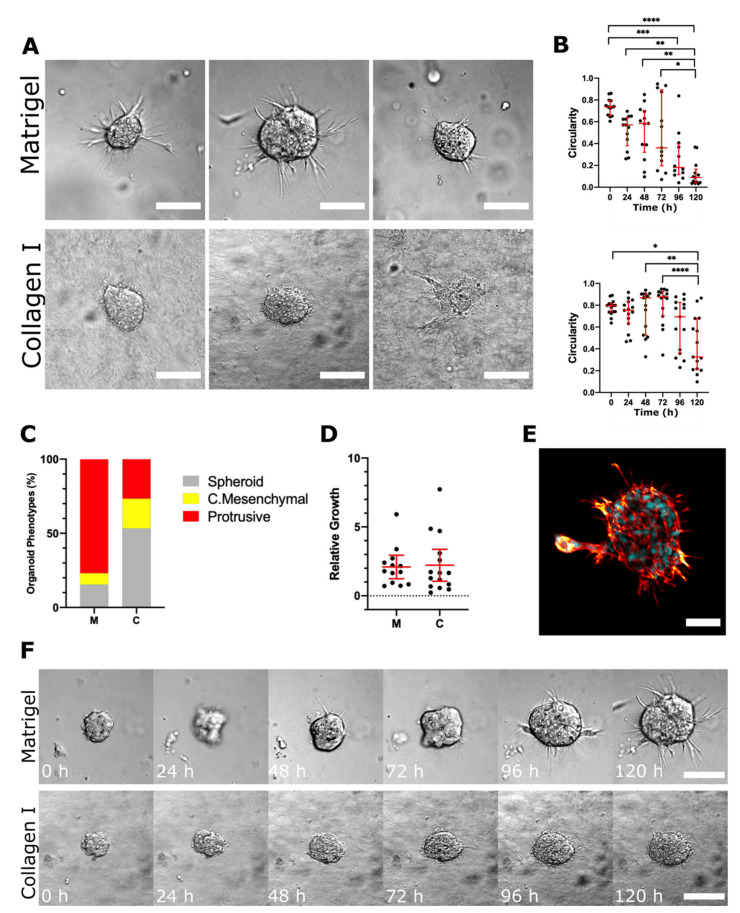
The invasive behaviour of SH-SY5Y cells is altered in response to an in vivo tumour microenvironment. (**A**) Representative DIC images of organoids isolated from SH-SY5Y xenografts after 5 days of culture in Matrigel and collagen. (**B**) Organoid circularity, where a value of 1.0 indicates a perfect circle, was measured to support microscopic observations of phenotypical changes in Matrigel and collagen (Kruskal–Wallis and Dunn’s post-hoc tests; horizontal bars represent median and interquartile range). (**C**) Bar charts represent the proportion of organoids per phenotype classification. (**D**) The relative growth of organoids was calculated by dividing the difference in area between 0 h and 96 h by the area at 24 h (Brown–Forsythe ANOVA with T3 Dunnett post-hoc tests; horizontal bars represent mean ± 95% confidence intervals). (**E**) Representative confocal image of a “protrusive” SH-SY5Y organoid in Matrigel; nuclei stained with DAPI (cyan) and F-actin stained with phalloidin (red). (**F**) Representative DIC time-lapse images of protrusive invasion in Matrigel and collagen I over a 120 h period. In all plots, each dot represents one organoid (*n* = 13 in Matrigel, *n* = 15 in collagen) and asterisks indicate statistical significance (* *p* < 0.05, ** *p* < 0.01, *** *p* < 0.001, **** *p* < 0.0001). DIC scale bars = 100 μM, confocal scale bars = 50 μM.

Lastly, we explored whether the in vivo tumour microenvironment is sufficient to induce more diverse invasion strategies in NB cells. We produced SH-SY5Y cell xenografts and isolated organoids from the resulting tumours for our in vitro 3D invasion assays. The behaviour of these organoids was distinct (Figure 6A) from that of the SH-SY5Y cell clusters discussed above (Figure 4). In Matrigel, the majority of SH-SY5Y organoids isolated from the PDXs (76%) resembled the protrusive phenotype (Figure 6A,C–E). Unlike the cell clusters, SH-SY5Y organoids were predominantly non-invasive in collagen, where 53% of organoids formed spheroids with no protrusions. However, we did also observe invasive organoids that employed both the protrusive and collective mesenchymal strategies of invasion in collagen. Measuring circularity confirmed a greater proportion of invasive organoids in Matrigel (Figure 6B); however, invasive organoids appeared less aggressive in collagen (Figure 6F). Overall, the in vivo tumour microenvironment promoted a distinct local invasion strategy of SH-SY5Y cells.

## 3. Discussion

### 3.1. NB Organoids Isolated from PDXs Are Phenotypically Heterogenous during Local ECM Invasion 

**Figure 7:**
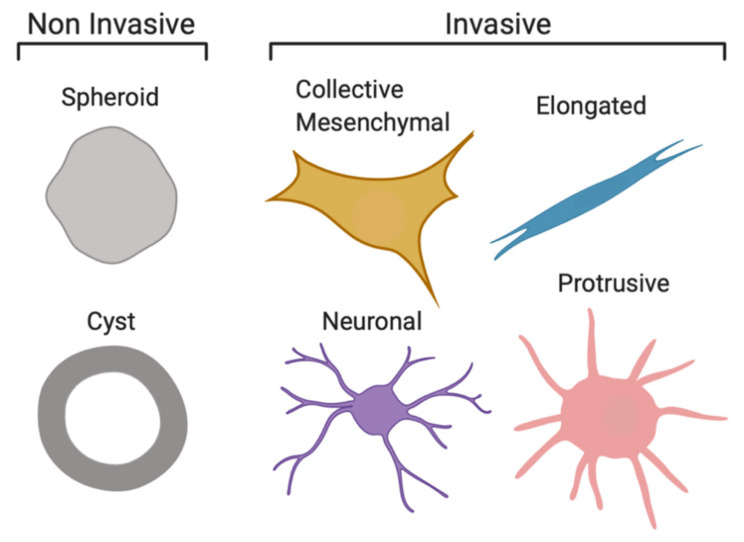
Schematic illustration of distinct morphological phenotypes observed in 3D invasion assays. Six distinct phenotypes in NB organoids isolated from PDXs were identified. Round organoids with no protrusions were termed “spheroid” and round organoids containing a lumen “cyst” due to their cyst-like appearance. Cells invading as collective strands with actin-rich protrusions (indicative of cells with mesenchymal traits) at the leading edge displayed a “collective mesenchymal” phenotype. These organoids contained strands invading in multiple directions. The “elongated” phenotype also displayed collective strand invasion, although multicellular streaming was present as well. Cells also migrated in a common direction leading with long actin-rich protrusions. “Neuronal” organoids invaded collectively as a dispersed network with transient cell–cell contacts; protrusions were neurite-like. The “protrusive” phenotype displayed a radial pattern of neurite-like protrusions, where cells migrated individually or were followed loosely by multicellular streams.

NB cells employed distinct strategies to invade ECM-mimicking hydrogels (Figure 7). We identified four invasive phenotypes based on morphological changes over time. NB organoids commonly invaded as collective strands maintaining adhesions between neighbouring cells. Cells at the leading edge of these strands appeared mesenchymal-like due to the presence of one or several actin-rich protrusions, indicative of cells with mesenchymal traits and a “collective mesenchymal” invasion. Previous studies have described similar modes of multicellular migration where leading cells proteolytically degrade the ECM, creating a track that is widened by following cells [18]. Collective strands have been reported for breast cancer cells in 3D in vitro and in vivo models [17,19], for melanoma cells using intravital imaging [20] and clusters of circulating tumour cells have been identified in patients with metastatic disease [33]. In a small study of 28 NB patients, one circulating tumour cell cluster was identified in a high-risk patient with distant lymph node metastasis [34]. We also observed collective strand invasion in our “elongated” organoids; however, this phenotype was molecularly distinct because multicellular branching is uncommon, and cells migrated in a common direction with a leading multicellular strand. In comparison, collective mesenchymal organoids extend multicellular strands in multiple directions, and these strands commonly branch.

NB is derived from neural progenitor cells and displays neuronal behaviour; neurite formation is commonly seen in NB cells [21,35]. We identified “neuronal” organoids with dispersed cells extending neurite-like branches into the matrix. This strategy of invasion is morphologically comparable to that seen in glioma, where cells invade as a collective network with transient cell–cell interactions in 3D in vitro and in vivo models [22,23]. We also found “protrusive” organoids that maintained a round central cell mass while radially extending long thin actin-rich protrusions into the matrix, which may be unique to NB organoids. These protrusions often lacked nuclei, and when nuclei were present, they were individually migrating or loosely followed by multicellular streams. This phenotype may also be neuronal due to the long leading protrusion that is neurite-like and can be compared to organotypic cultures of glioblastoma that also display a radial pattern of invasion [36]. That being said, glioblastoma invasion appears less directional than that described here, and single cell dissemination was common in that model, while disseminating cells were only occasionally detected in NB organoids.

Phenotypic heterogeneity was also seen in non-invasive organoids (Figure 7). “Spheroid” organoids maintained a round cellular mass, while “cyst” organoids developed a lumen-like structure. Cysts were commonly found in Matrigel, suggesting that Matrigel also promotes diversity amongst non-invasive NB organoids. Cyst formation was described as a bi-layered epithelial structure that models the formation of epithelial ducts [25]. Although we did not determine whether the cyst-like structures were bi-layered, we speculate, given the origin of NB, that they may be in fact neuroepithelial in nature. Neuroepithelial cyst formation in 3D cultures was reported as a model of neural tube development in vivo [26]. Our results suggest that the intratumour heterogeneity of NB is retained within a PDX, with neuroepithelial, neuronal or mesenchymal cell populations. However, the proposed terminology will need to be confirmed in the future by mapping these categories to differing molecular pathways or histopathological observations.

### 3.2. The Phenotypic Heterogeneity Seen in PDX Organoids Was Not Recapitulated in These Cell Lines

NB cell lines grown in the same platform did not display cellular heterogeneity as seen in PDX organoids. NB cell lines tested herein may have intrinsic phenotypes that determine their migratory behaviour. EMT is an “intrinsic cell autonomous process” or a transient process in response to environmental stimuli [37]. Intrinsic EMT and stem cell-like phenotypes can be made irreversible due to genetic alterations [38]. The in vivo tumour microenvironment induced a distinct organoid phenotype in SH-SY5Y cells. SH-SY5Y cells were taken directly from a 2D monolayer culture where they were homogenously exposed to environmental factors. When we introduced SH-SY5Y cells to an in vivo tumour microenvironment, they grew as a tumour mass, which was heterogeneously exposed to such factors. In a hypoxia-sensing xenograft model, which separates hypoxic cells from their non-hypoxic counterparts, a distinct phenotype in hypoxic compared to non-hypoxic breast cancer cells was detected without depriving cells of oxygen in vitro [39]. The in vivo growth of cell lines (xenograft) was also linked to phenotypical changes in tumour-initiating cells in ovarian cancer [40]. We conclude that the in vivo tumour microenvironment increases the cellular heterogeneity in NB cell lines, but the selection for growth in vitro over many generations limits their invasion strategies.

### 3.3. Matrigel Is the Preferred Substratum for NB Invasion

We adapted an organoid model described for murine and human primary and metastatic breast tumours to study local NB cell invasion [41]. We grew organoids from NB PDXs in ECM hydrogels to monitor the dynamics of cell invasion in real-time. Collagen type-I gels but not Matrigel has been shown to promote the invasion and dissemination of malignant mammary epithelial cells [41,42]. Furthermore, in pancreatic cancer [43] and colon cancer, collagen type-I gels induce the expression of EMT genes [44]. Conversely, we showed that NB organoids preferentially invaded Matrigel.

During development, ECM components are classified into three distinct categories: permissive, non-permissive and inhibitory. Permissive ECM components, including laminin, fibronectin, collagen type-I and type-IV, are expressed along the migratory pathways of neural crest cells, the origin of NB, and promote cell motility. Certain laminin isoforms, fibronectin and collagen type-IV, are critical for the development of the neural crest [45]. NB cells migrate extensively in Matrigel, which contains these ECM molecules [27], which is in line with our findings.

The migration of neural crest cells is optimal in the basement membrane compared to collagen type I gels, and a competitive agonist for laminin (YIGSR) inhibits crest cell migration in Matrigel, suggesting that laminin is essential for neural crest cell motility [46]. Laminin-containing basal laminae are the preferred substratum for the migration of neural crest cells [47]. Laminin is also a promoter of neurite formation [48]. Thus, we speculate that the embryonal origin of NB may be explained by its preferential invasion of the laminin-rich matrix, Matrigel.

### 3.4. Local Invasion of NB Cells Is Dependent on the Sample of Origin

We showed that organoids isolated from 603x and Felix displayed an aggressive invasive behaviour in our ECM-mimicking cultures, in particular in Matrigel. In contrast, organoids isolated from 573x and 424x were non-invasive in both matrices tested. The NMA PDX, Felix, yielded organoids that displayed the most aggressive invasion. This may be due to the selection processes that occurred in the patient over multiple courses of therapy and the origin of the PDX, circulating tumour cells obtained post-mortem. The other three PDXs were established from samples taken at the time of diagnosis, prior to therapy. Clonal clusters derived from the NMA-amplified cell line, SH-SY5Y, and organoids derived from SH-SY5Y tumours also showed an aggressive invasion. As both a *MYCN* non-amplified cell line and a PDX displayed an aggressive behaviour, factors other than *MYCN* amplification may promote NB cell invasion. This is consistent with the large number of patients having high-risk non-*MYCN*-amplified tumours that present with metastatic disease [49]. Our 3D model of local cell invasion is a valuable tool to study NB biology. By pairing NB organoids and 3D invasion assays, we may uncover genes and pathways that are involved in the local invasion of NB cells.

### 3.5. Repression of MYCN Transcription Promotes Less Aggressive Cell Behaviour

The amplification of MYCN is an unfavourable prognostic factor in NB and correlates with aggressive disease and worse outcome [31]. As expected, we found that MYCN-expressing cell clusters grew larger and displayed a more aggressive invasion. The literature supports our finding that MYCN expression is critical in the local invasion of NB cells. In transwell migration and invasion assays, higher levels of MYCN expression were correlated with increased cell motility and invasion [50]. In high-risk NB, upregulation of the EMT-promoting transcription factor TWIST1 was correlated with MYCN amplification and a MYCN transcriptional target [51]. MYCN has been shown to repress the transcription of the death receptor antagonist, Lifeguard (FAIM2), which is downregulated in high-risk NB. Repression of Lifeguard decreased cell adhesion and increased cell motility, promoting a more invasive phenotype [52]. Thus, MYCN transcription regulates aggressive cell behaviour.

### 3.6. Benefits of 3D NB Cell Assays over Current Models of Local Invasion

Our 3D model has many benefits over current models of local invasion, both in NB and other cancer types. Studies using organoid assays are relatively affordable, particularly when compared to murine models of metastasis. Our model offers superior optical accessibility and higher resolution imaging than in vivo models of local invasion. Local cell invasion was observed within a couple of days, another advantage over long-term in vivo studies. Although the development of PDXs can take some time, once established, using PDX-derived organoids greatly improves their experimental yield. There are many established NB PDXs available [10,15,53].

NB invasion in vitro has been mostly characterised by the scratch and transwell assays. We performed 3D invasion assays with NB cell lines to compare their behaviour to that observed in NB organoids and to published studies using transwell assays [54,55]. Cells were serum-starved for 24 h and transwells coated with basement membrane extract. Shankar’s study included three of the cell lines used herein, namely, Kelly (NB 19), SHEP and Lan-1 [54]. In our 3D model, we observed highly aggressive behaviour for SHEP and Lan-1 cells, while Kelly cells were non-invasive. Conversely, Shankar et al. described Kelly cells as highly invasive and SHEP and Lan-1 cells as less invasive, with an invasion index of 7 for Kelly cells and invasion indices of <1 for SHEP and Lan-1 cells. The stark contrast in cell behaviour between both assays may be due to the difference in dimensionality and the fact that our 3D model enables cells to migrate collectively. Differences in cell behaviour between 2D and 3D cultures are well known [56,57]. NB cells grown in 3D scaffolds display a 100-fold increased resistance to cisplatin compared to cells grown as 2D monolayers [58]. This chemotherapeutic resistance is comparable to that seen in orthotopic xenografts, highlighting the physiological relevance of 3D models. The phenotype and motility of glioma cells have been compared in 2D and 3D [59]. In 2D, glioma cells displayed a sheet-like morphology, non-directional migration and formation of random lamellipodium. Glioma cells invading 3D collagen gels were phenotypically similar to neural-progenitor cells, with a round cell body and a long leading process. This motility pattern was confirmed by cells grafted into organotypic brain slice cultures, suggesting that invasion in 3D closely resembles the in vivo scenario. Cellular heterogeneity among invasive cells was detected in both collagen gel and brain slice cultures but was absent in 2D.

Our results show that NB cells employed a range of distinct migration strategies. Notably, NB cells often migrated collectively as strands (collective mesenchymal) or as streams (protrusive, neuronal). However, transwell assays only assess the migration/invasion of individual NB cells. There is a lack of literature related to the modes of migration/invasion in NB in vivo, but some studies have assessed the migration of neural crest and melanoma cells. In the embryo, neural crest cells migrate as dense cohorts of cells that depend on three factors for directed migration: contact inhibition of locomotion, co-attraction and confinement [60]. Stream migration relies on leader cells at the invasive front with specific molecular signatures [61]. The presence of leader cells and the maintenance of cell–cell contacts are documented as requirements for trunk migration of neural crest cells [62]. In a process known as contact-stimulated migration, both neural crest and melanoma cells migrate far more effectively as a cohesive group [63]. Due to its origin, we suspect that collective cell migration may be the preferred mode of motility in NB. When modelling NB cell invasion, collective migration must be enabled, and thus, our 3D model is superior to the transwell assay. Our 3D model is more indicative of the in situ scenario than currently used 3D in vitro spheroid models of NB invasion [64]. In spheroids, cells do not display directional, multicellular and invasive structures. In fact, cell proliferation contributes largely to the spreading of spheroids. The phenotypic heterogeneity we observed during local invasion of organoids offers an advantage over current invasion assays.

Our 3D model also has some limitations. Given its origin, Matrigel varies from batch-to-batch. Its exact constituents are not well defined and may impact the signals that promote the observed cell behaviour [65]. Matrigel may not represent the human NB microenvironment. However, there is a lack of literature regarding the ECM composition in both NBs and healthy human embryos.

Our 3D model lacks the stromal tumour microenvironment. Cancer-associated fibroblasts (CAFs) and immune cells facilitate tumour cell invasion through ECM remodelling, physical interactions with cancer cells and pro-invasive stimuli [66,67]. In various co-culture model systems, both CAFs and macrophages have been shown to promote the invasion and migration of tumour cells from many cancer types [16,68,69,70,71,72,73]. In future studies, further optimisation of our 3D model is required to facilitate 3D co-cultures. Although this will add a level of complexity to our assays, we will be able to explore the roles of stromal cells in local NB cell invasion.

In conclusion, we characterised six distinct phenotypes in organoids derived from human NB. In future studies, we will confirm that these phenotypes are molecularly distinct by profiling the differential gene expression and invasive gene signatures. Our 3D culture model is suited for the study of NB biology, including the impact of gene knockdown/induction on local matrix invasion.

## 4. Materials and Methods

### 4.1. NB Cell Lines and Culture Conditions

The neuroblastoma cell lines LAN-1, Kelly, CHP212, SH-SY5Y and SH-EP Tet21N [32], doxycycline-repressible (Tet-Off) MYCN gene cells, were cultured in corresponding cell culture media (Table S2.1 in File S2) containing 10% heat-inactivated FBS (#F0926, Sigma-Aldrich, St. Louis, MO, USA) with 50 U of penicillin per mL and 0.1 mg of streptomycin per mL at 37 °C in a 5% CO_2_ humidified atmosphere. All cell lines were authenticated by DNA profiling before use.

### 4.2. Patient-Derived Xenograft Generation and Harvesting

Neuroblastoma-specific PDX tumours, COG-N-424x, COG-N-573x, COG-N-603x and COG-N-Felix, were established in the laboratory of Dr. C. Patrick Reynolds the Children’s Oncology Group (COG) and ALSF Childhood Cancer Repository (Sciences Center Texas Tech University Health, 2018). Briefly, selected PDXs were subcutaneously flank-engrafted into immuno-deficient mice (NOD.CB17-Prkdcscid/SzJ, NOD/SCID) and grown until tumours reached 1500 mm^3^, and tumours were harvested and cryopreserved with DMSO.

SH-SY5Y cells were subcutaneously xenografted into 6–8 week old female Hsd:Athymic Nude-Foxn1nu mice and grown until tumours reached 1000 mm^3^.

### 4.3. Isolation of Neuroblastoma PDX Organoids

NB PDX organoids were isolated using previously described techniques [41]. Briefly, the PDX tumours were minced with a scalpel and digested into tumour fragments by a combination of mechanical disruption and collagenase/trypsin digestion. These fragments were separated by differential centrifugation to remove both single cells and incompletely digest fragments. The final pellet was composed of similar size tumour fragments, each containing 30–60 cells, which we termed “organoids” (Figure S1.1 in File S1). NB organoids were assessed in the presence of murine stroma by co-immune fluorescence staining with DAPI and a neuroblastoma-specific marker using an anti- N-Myc (D1V2A) Rabbit mAb (#84406, Cell Signaling Technology, Beverley, MA, USA) followed by labelling with Alexa Fluor^®^ 488 Goat anti-Rabbit IgG secondary antibody (Figure S1.6 in File S1).

### 4.4. Neuroblastoma Organoid Culture

NB PDX-derived organoids or single cell suspensions were embedded in 3D Matrigel (354230; BD Biosciences) or rat-tail collagen I (#354236; Corning) using 24-well black coverslip-bottomed plate (#662892, Greiner Bio-One). Acid-solubilized rat-tail collagen I gels (3 mg/mL collagen I, pH 7–7.5) were prepared as described in [41]. For each matrix, organoids or single cells were mixed to yield a suspension of two or three organoids or cells/μL. Then, a 100-μL mix was plated in each well on a 37 °C heating block, followed by incubation at 37 °C for 45 min to allow polymerisation. Tumour organoids were cultured in 1 mL of 2.5-nM FGF2 in organoid media (DMEM/F12, 50 U of penicillin per mL, 0.1 mg of streptomycin per mL, 1% insulin-transferrin-selenium). Cell clusters were cultured in a corresponding complete media (Table S2.1 in File S2).

### 4.5. Time-Lapse Differential Interference (DIC) Contrast Microscopy

NB PDX organoids or individual cells were live imaged using a LD Plan-Neofluar 20×/0.4 Korr Ph2 objective lens, a Zeiss AxioObserver Z1 and an Axio- Cam MRM camera. In general, images were captured every 20 min for 5 days beginning on the day 0 organoids were plated (0 h) maintaining temperature at 37 °C and CO_2_ at 5%. Cell line clusters were monitored and visualized daily after initial cell seeding. Some of the movies of NB PDX were collected using a Zeiss Axiovert S-100 microscope and a Cohu CCD camera, as previously reported [41]. Temperature was held at 37 °C and CO_2_ at 5%.

### 4.6. Quantification of Organoid Growth and Invasion

Growth and invasion of organoids and cell clusters were quantified by manually outlining their perimeters from DIC images and measuring the circularity with ImageJ (National Institutes of Health) (File S2). For growth, paired images for each organoid were obtained at 0 and 24 h after plating, and growth was represented as a fold change in projected area in 24 h.

### 4.7. Fluorescence Staining of 3D Hydrogels

Once the imaging experiments were completed (120 h in culture), samples were washed with DPBS, fixed in 2% paraformaldehyde (PFA), permeabilized with 0.5% Triton X-100 and immediately blocked with 10% FBS in DPBS. The samples were then incubated with phalloidin and DAPI to stain F-actin and cell nuclei, respectively. Images of organoids/cell clusters were taken using a laser-scanning confocal microscope (Zeiss LSM780, Jena, Germany).

### 4.8. Statistical Analysis

All statistical analyses were performed using Prism Software (GraphPad Software Inc, La Jolla, CA, USA). The relative growth of organoids/cell clusters across different matrices and different conditions was compared. This analysis involved comparing unmatched Gaussian populations where equal variances could not be assumed. Therefore, we used either unpaired *t*-tests with Welch’s correction or Brown–Forsythe One-Way ANOVAs followed by T3 Dunnett post-hoc tests, depending on the number of groups, to compare the equality of means between populations. Statistical analyses were performed to compare the circularity of organoid/cell cluster populations at different time points. In this case, we had non-normally distributed data and thus performed non-parametric analyses using Kruskal–Wallis followed by Dunn’s post hoc for multiple comparison between time-points. A value of *p* < 0.05 was considered statistically significant.

## 5. Conclusions

This work demonstrates that 3D invasion assays are a suitable tool to study local invasion in NB. Various matrix compositions induced distinct cell behaviours, with Matrigel being the preferred substratum for local organoid invasion. Organoid invasion was PDX- and cell line-dependent and divided into six distinct phenotypes in PDX-derived organoids. In contrast, NB cell lines were phenotypically confined during invasion of the local ECM, while organoids isolated from cell line-derived xenografts displayed a broader range of phenotypes compared to clonal cell line clusters. The addition of FBS and bFGF induced the most aggressive cell behaviour and the widest range of phenotypes. In contrast, the repression of the prognostic NB marker, *MYCN,* resulted in less aggressive cell behaviour. By pairing PDX-derived organoids and 3D invasion assays with high-resolution and real-time analysis, the molecular mechanisms that control local invasion are uncovered.

## Figures and Tables

**Table 1:** Characteristics of NB PDXs used.

PDX	Risk Classification	Phase of Therapy	Sample Type	Stage	*MYCN* Status	*ALK* Status	*TERT* mRNA	Ref.
COG-N-424x	High	DX	Tumour	4	Amp	WT	++	[15,28]
COG-N-573x	High	DX	BM	4	Amp	WT	++	[28]
COG-N-603x	High	DX	Tumour	4	Amp	WT	+++	[15,28,29]
Felix-PDX	High	PD-PM	Blood	4	Non-Amp	ALK F1245C	++	[15,28,30]

DX, diagnosis; BM, bone marrow; PD, progressive disease; PM, post-mortem; Amp, amplified; WT, wild type; +, low; ++, intermediate; +++, high.

**Table 2:** The approximate timepoints at which NB invasion started in Matrigel and collagen matrices.

Cell Line/Organoid	Time	(Hours)
	Matrigel	Collagen
SHEP-21N(MYCN-On/Off)	<24	24–48
LAN-1	<24	48–72
SH-SY5Y	24–48	48–72
SH-SY5Y Organoids	<24	48–72
COG-N-573x	<48	<12 *
COG-N-603x	<48	<12 *
COG-N-Felix	<48	>120 *

* primarily non-invasive.

## Data Availability

Data is contained within the article or Supplementary Materials. The original raw image data are available upon request from the corresponding author olgapiskareva@rcsi.com.

## Supplementary Materials

The following are available online at https://www.mdpi.com/2072-6694/13/4/736/s1, File S1: Growth and invasion of PDX organoids, File S2: Materials and Methods.
