# Supplementary material for: Neuroblastoma Invasion Strategies Are Regulated by the Extracellular Matrix
Source: Cancers (Basel). 2021 Feb 10;13(4):736. doi: 10.3390/cancers13040736 (PMC7916632; doi:10.3390/cancers13040736)
Supplement: Supplementary file 1 [file cancers-13-00736-s001.zip › cancers-1084564-suppl-XML/cancers-1084564-File S1-XML.docx]

**File S1: Growth and invasion of PDX organoids**

**Figure S1.1.** The size of isolated NB organoids depends on the PDX of origin. Organoids were isolated from four different NB PDXs and embedded in Matrigel or Collagen hydrogels. Images of organoids were captured by DIC microscopy at t0hr and area was measured using FIJI image analysis software. Each dot represents one organoid and error bars indicate mean ± 95% confidence interval. Asterisks indicate statistical significance obtained using Brown-Forsythe ANOVA with T3 Dunnett post-hoc test (* *p* < 0.05, ***p* < 0.01, *** *p* < 0.001).


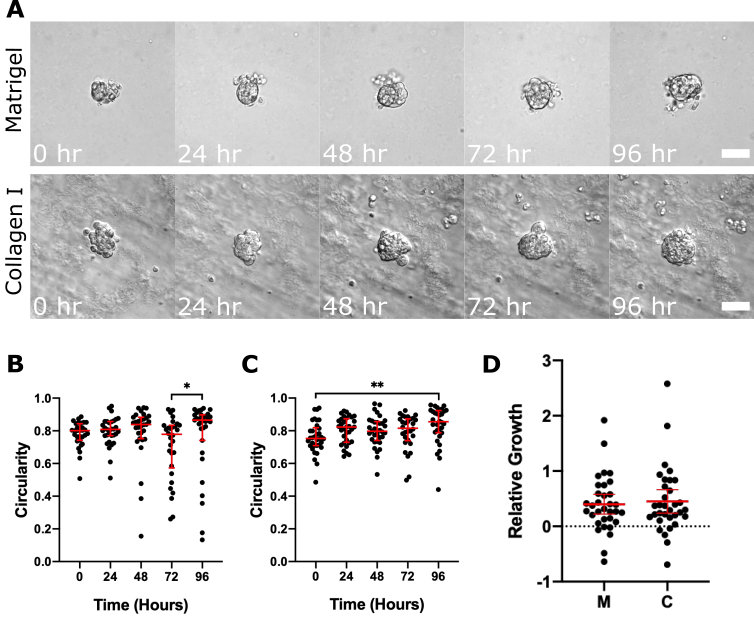


**Figure S1.2** Organoids isolated from the COG-N-424x PDX are non-invasive in 3D hydrogel cultures. Representative DIC time-lapse images of spheroid organoids in 3D M and C hydrogels (**A**). Measurements of organoid circularity over five days confirms no significant phenotype changes (a value of 1 represents a perfect circle) in Matrigel (**B**) or Collagen I (**C**) gels (Kruskal-Wallis and Dunn’s post-hoc tests, horizontal bars indicate median and interquartile range). The relative growth of organoids was calculated by dividing the difference in area between t0hr and t96hr by the area at t0hr and presented on the plot (**D**) (Unpaired t test with Welch’s correction, horizontal bars indicate mean ± 95% confidence interval). For all plots, each dot represents one organoid (*n* = 32 in M, *n* = 35 in C) and asterisks indicate statistical significance (* *p* < 0.05, ** *p* < 0.01). All scale bars = 50μm.


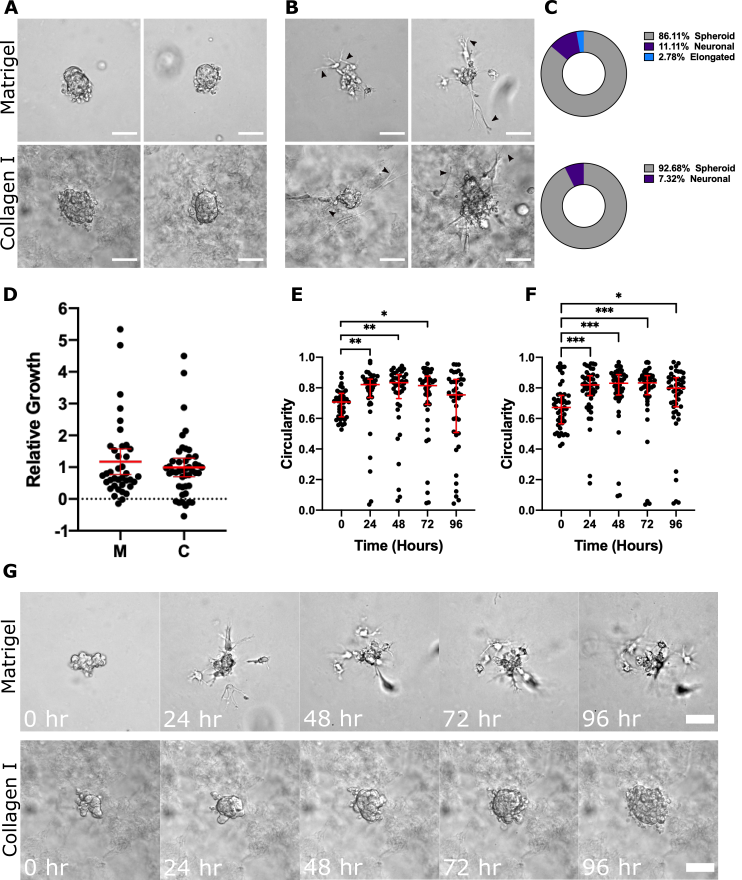


**Figure S1.3** Organoids isolated from 573x are predominantly non-invasive and the small proportion of invasive organoids favour the neuronal phenotype. Representative DIC images of organoids isolated from 573x in 3D M or C hydrogels. Organoids were characterised as non-invasive spheroids (**A**) or invasive neuronal organoids (**B**), representative images were taken at t96hr hours and t24hr hours in culture respectively. Pie-charts represent the proportion of organoids per phenotype classification (**C**). The relative growth of organoids was calculated by dividing the difference in area between t0hr and t96hr by the area at t0hr and presented on the plot (**D**) (Unpaired t test with Welch’s correction, horizontal bars indicate mean ± 95% confidence interval). Measurements of organoid circularity, where a value of 1 indicates a perfect circle, confirm microscopic observations of changes in morphology in M (**E**) and C (**F**) hydrogels (Kruskal-Wallis and Dunn’s post-hoc tests, horizontal bars indicate median and interquartile range). **G**) Representative DIC time-lapse images of a neuronal organoid invasion in M and spheroid formation in C. Black arrowheads indicate neurite-like processes. For all plots, each dot represents one organoid (*n* = 41 in M, *n*=46 in C) and asterisks indicate statistical significance (* *p* < 0.05, ** *p* < 0.01, *** *p* < 0.001, ns; not significant). All scale bars = 50μm.


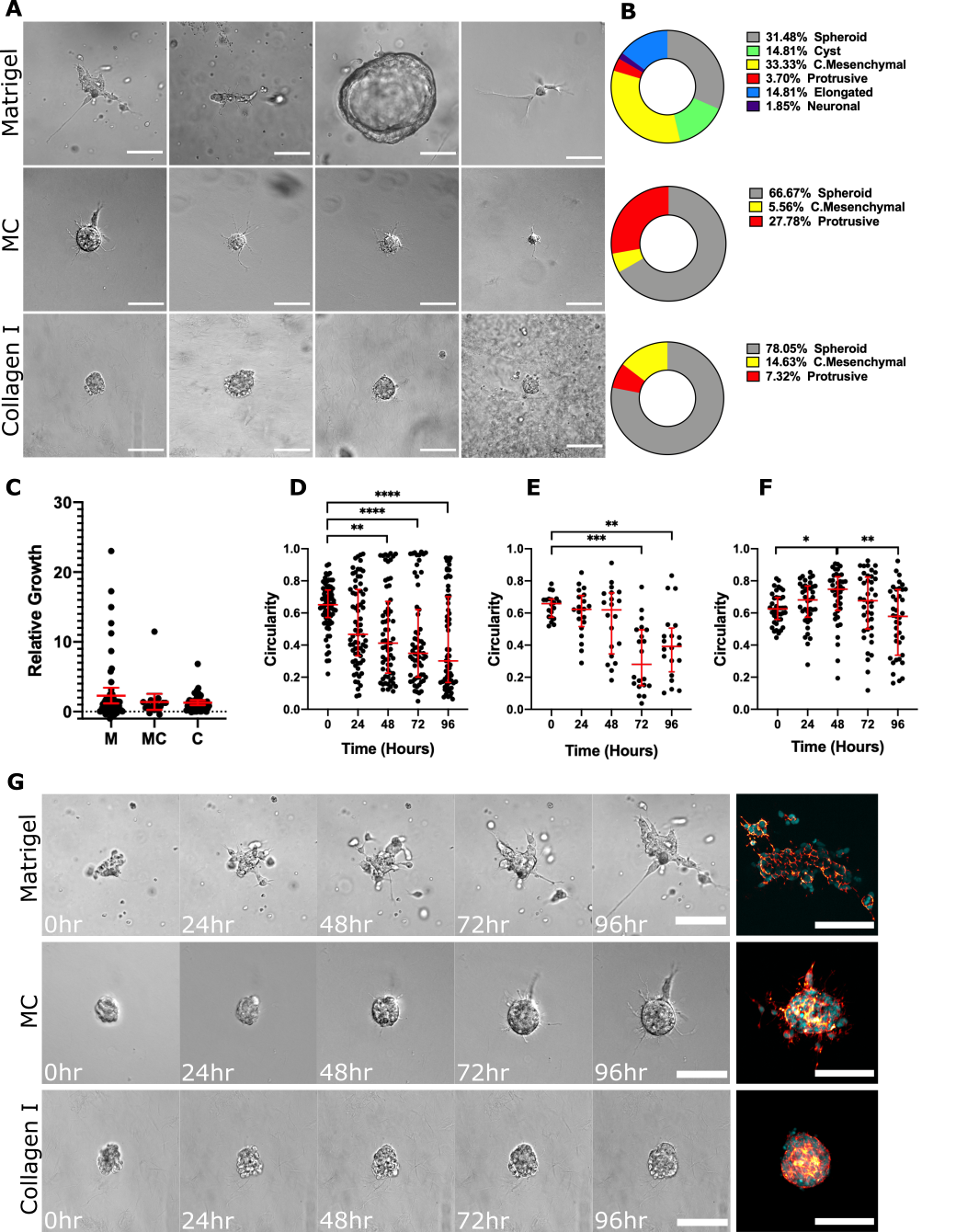


**Figure S1.4.** The local invasion of organoids isolated from 603x is dependent on ECM composition. Representative DIC images of NB organoids after 4 days of culture in 3D hydrogels composed of M, MC or C (**A**). Pie-charts represent the proportion of organoids per phenotype classification (**B**). The relative growth of organoids was calculated by dividing the difference in area between t0hr and t96hr by the area at t0hr and presented on the plot (**C**) (Brown-Forsythe and Welch ANOVA with T3 Dunnett post-hoc test, horizontal bars indicate mean ± 95% confidence interval). Organoid circularity was measured to support microscopic observations of phenotypical changes, where a value of 1.0 indicates a perfect circle, in M (**D**), MC (**E**) and C (**F**) hydrogels (Kruskal-Wallis and Dunn’s post-hoc tests, horizontal bars indicate median and interquartile range). (**G**) Representative DIC time-lapse and confocal images of prevalent organoid phenotypes in each of the respective matrices; collective mesenchymal in M, protrusive in MC and spheroid in C. Nuclei are stained with DAPI (cyan) and F-actin is stained with phalloidin (red hot). For all plots, each dot represents one organoid (*n* = 63 in M, *n* = 20 in MC, *n* = 40 in C) and asterisks indicate statistical significance (* *p* < 0.05, ***p* < 0.01, *** *p*< 0.001, **** *p* < 0.0001). All scale bars = 100μm.


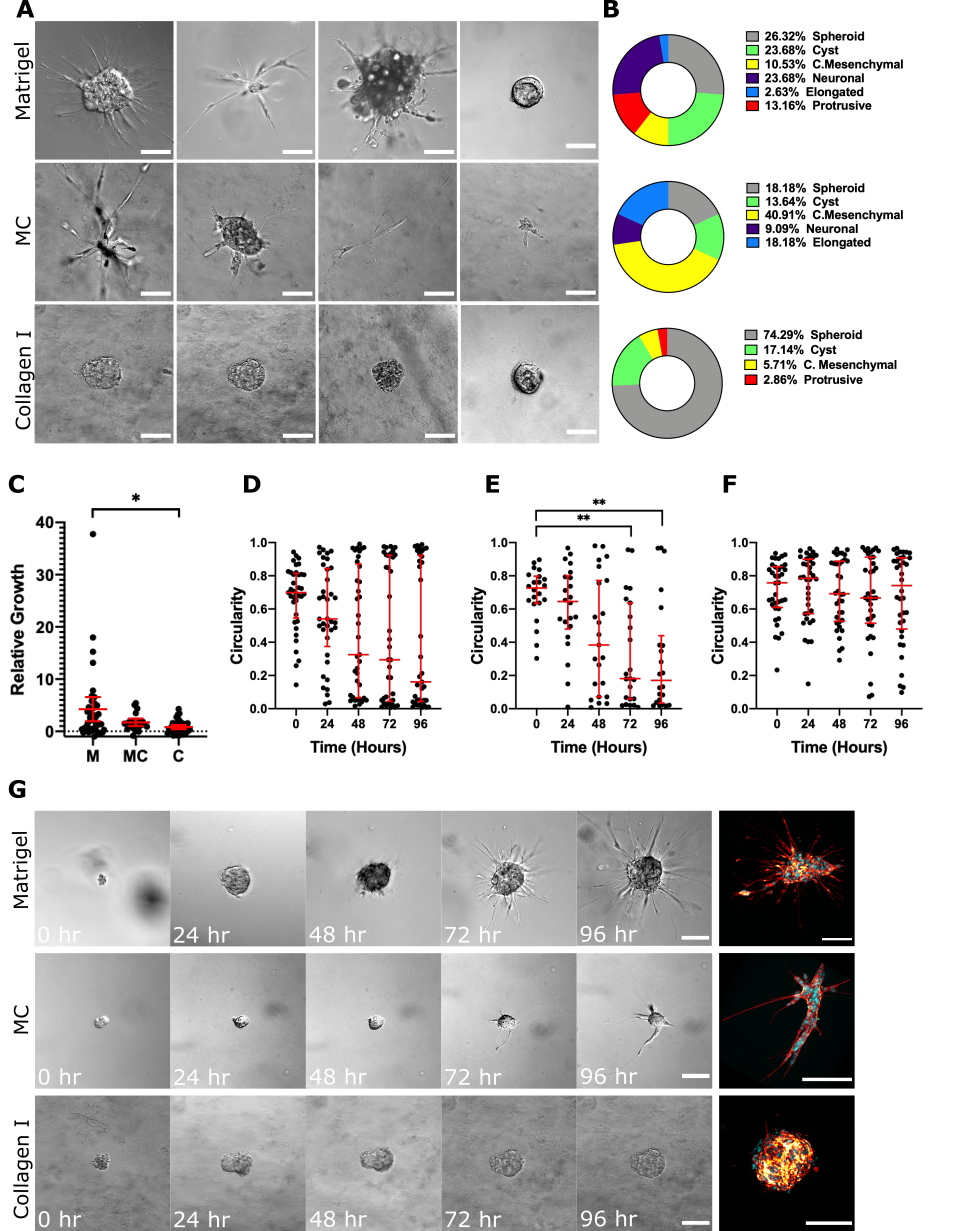


**Figure S1.5.** Invasion of organoids isolated from Felix is dependent on the composition of the matrix. Representative DIC images of organoids isolated from Felix taken at t96hrs in 3D M, MC, or C hydrogels (**A**). Pie-charts represent the proportion of organoids per phenotype classification (**B**). The relative growth of organoids was calculated by dividing the difference in area between t0hr and t96hr by the area at t0hr and presented on the plot (**C**) (Brown-Forsythe and Welch ANOVA with T3 Dunnett post-hoc test, horizontal bars indicate mean ± 95% confidence interval). Plotting the circularity of organoids, where a value of 1.0 indicates a perfect circle, over time supports our microscopic observations of phenotype alterations in M (**D**), MC (**E**) and C (**F**) (Kruskal-Wallis and Dunn’s post-hoc tests, horizontal bars indicate median and interquartile range). Representative DIC time-lapse and confocal images of prevalent organoid phenotypes in each of the respective matrices; protrusive in M, collective mesenchymal in MC and spheroid in C. Nuclei are stained with DAPI (cyan) and F-actin is stained with phalloidin (red hot). For all plots, each dot represents one organoid (*n* = 38 in M, *n* = 22 in MC, *n* = 36 in C) and asterisks indicate statistical significance (* *p* < 0.05, ** *p* < 0.01, *** *p* < 0.001, **** *p* < 0.0001). All scale bars =100 μm.

Merged DAPI N-Myc F-actin


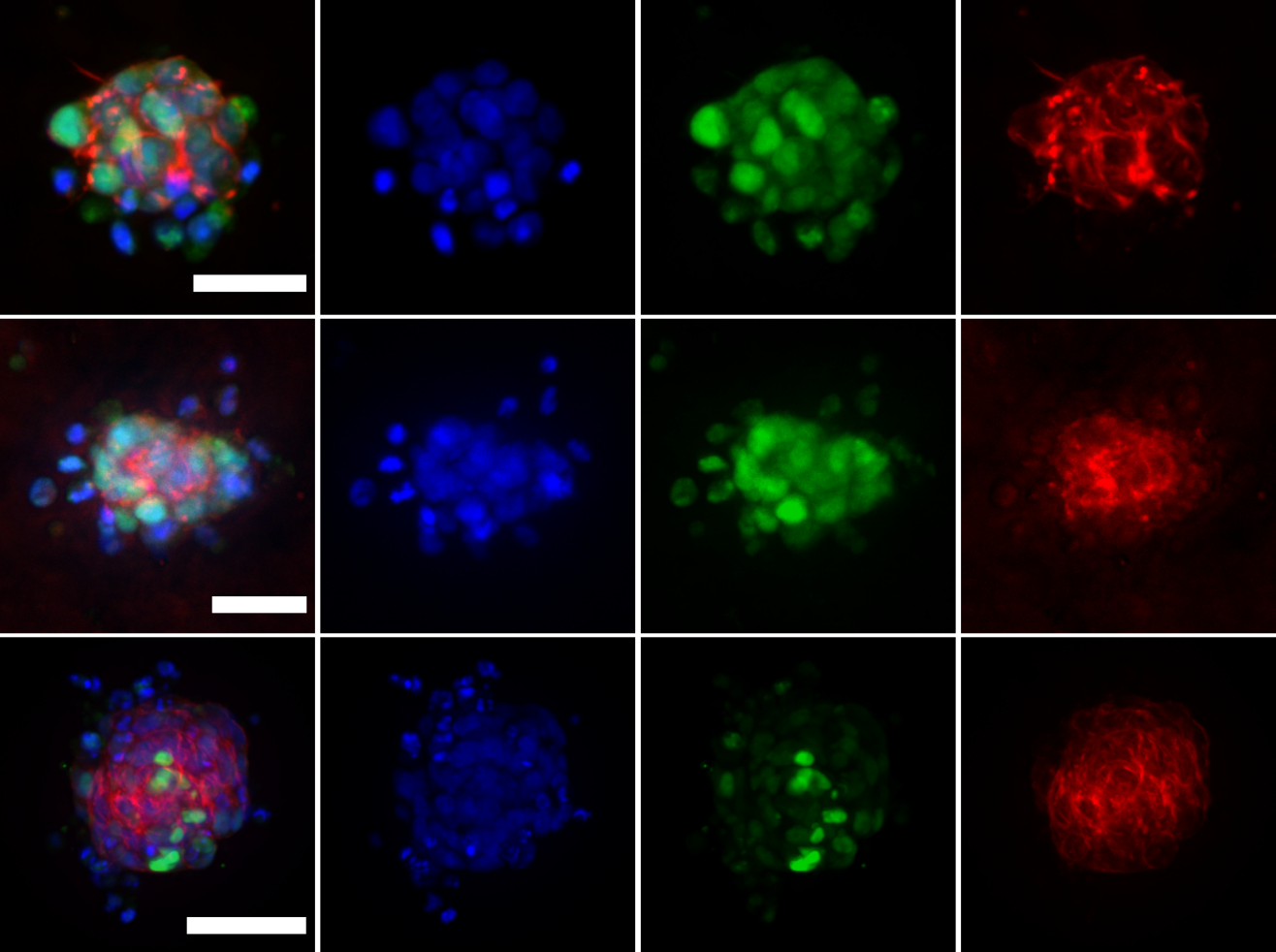


424x 603x 573x

**Figure S1.6**. Immunofluorescent analysis of neuroblastoma PDX organoids demonstrates nuclear localization of N-MYC. Neuroblastoma cells were labeled with an anti-N-Myc (D1V2A) Rabbit mAb (#84406, Cell Signaling Technology) and then tagged with Alexa Fluor® 488 Goat anti-Rabbit IgG secondary antibody (green). Nuclei are stained with DAPI (cyan) and F-actin is stained with phalloidin (red hot). All scale bars = 50 μm.
