# Supplementary material for: Neuroblastoma Invasion Strategies Are Regulated by the Extracellular Matrix
Source: Cancers (Basel). 2021 Feb 10;13(4):736. doi: 10.3390/cancers13040736 (PMC7916632; doi:10.3390/cancers13040736)
Supplement: Supplementary file 1 [file cancers-13-00736-s001.zip › cancers-1084564-suppl-XML/cancers-1084564-File S2-XML.docx]

**Supplementary File 2: Materials and methods**

**Table 2.1.** The neuroblastoma cell lines and PDXs used in this study.

| Cell Line/  PDX | MYCN Amplified | Chromosome 1p Alteration | Chromosome 17q Alteration | ALK Mutation | Culture Medium |
| --- | --- | --- | --- | --- | --- |
|  |  |  |  |  |  |
| Kelly | ✓ |  | gain | ALK F1174L | RPMI, 10% FBS, P/S |
| CHP212 | ✓ | loss | gain | WT | MEM:F12, 10% FBS, 1% L-glut, P/S |
| SHSY-5Y | ✕ | gain |  | ALK F1174L | MEM:F12, 10% FBS, P/S |
| SHEP-21N | repressible |  | gain | ALK F1174L | RPMI, 10% FBS, P/S |
| Lan-1 | ✓ | deletion |  | ALK F1174L | MEM:F12, 10% FBS, P/S |
| Felix-PDX | ✕ |  |  | ALK F1245C | DMEM/F12, P/S, 1% ITS |
| COG-N-424x | ✓ |  |  | WT | DMEM/F12, 1% P/S, 1% ITS |
| COG-N-573x | ✓ |  |  | WT | DMEM/F12, P/S, 1% ITS |
| COG-N-603x | ✓ |  |  | WT | DMEM/F12, P/S, 1% ITS |

FBS; fetal bovine serum, P/S; 50 U of penicillin per mL, 0.1 mg of streptomycin per mL, ITS, insulin-transferrin-selenium

## S2.1 FIJI image analysis

The DIC images were manually examined to classify organoid/cell cluster phenotypes based on morphology. NB organoids were grouped into six distinct phenotype classifications based on specific morphological traits. The images were then analysed and processed using FIJI (Fiji is just imageJ) image analysis software. Images were spatially calibrated and organoids/cell clusters were selected by manually outlining their perimeter (Figure S1).

The area of organoids/cell clusters was then calculated by FIJI software in μm^2^. By comparing the area of organoids/cell clusters every 24 hours their relative growth at any given time point could be calculated using the following formulas; *area t(x)-area t0hr/area t0hr* for organoids and *area t(x)-area t24hr/area t24hr* for clonal cell clusters. Using the same method for organoid and cell cluster selection, we also used FIJI to calculate circularity values, which we used to confirm our microscopic observations of phenotypic changes. This is calculated by FIJI using the following formula; *4π*area/perimeter ^2^*. Circularity is calculated as a fraction of 1.0, where the value 1.0 indicates a perfect circle, while values approaching 0.0 indicate an increasingly elongated shape. Circularity measurements were used to support microscopic observations of invasiveness, where decreasing circularity was found to correlate with increasing levels of invasion. Large organoids/cell clusters can generally be classified as invasive, mildly invasive or non-invasive based on the respective circularity scores; <0.4, 0.4–0.7, >0.7. It is important to note that circularity values lack validity for very small cells/clusters.


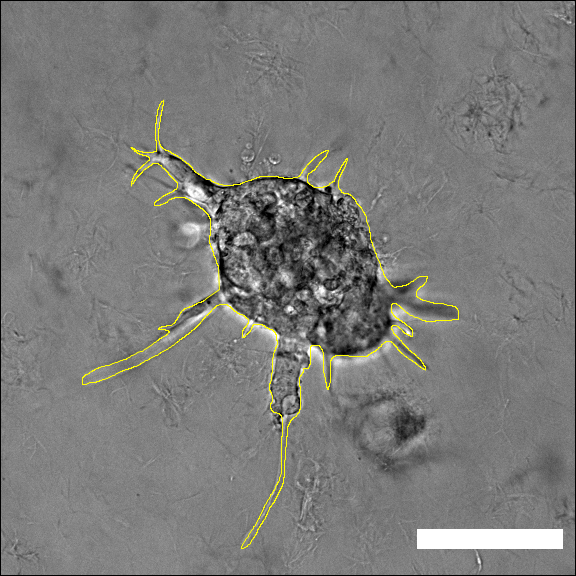


**Figure S2.1** Representative image of manual organoid selection using FIJI image analysis software. Example illustrating the manual selection of an organoid using FIJI image analysis software. Area = 16.37 × 10^3^μm ^2^, circularity = 0.084. Scale bare = 100μm

## S2.2. Exclusion Criteria

A number of technical issues were encountered while processing this dataset which led to certain organoids and/or cell line clusters being excluded from the analysis. A small proportion of organoids/clusters came into contact with and adhered to the cell culture plate, resulting in cells spreading out into a 2D monolayer (Figure S2.2 A). These organoids/cell clusters were easily identifiable due to their transparent appearance and their significant cell spreading that results in an unusually large size compared to populations that remain embedded in the gels. Non-viable organoids/cell clusters were also excluded from the analysis. These organoids/cell clusters maintained an identical morphology and size across time-lapse images indicating the absence of any viable cells (Figure S2.2 B). When organoids/cell clusters were embedded in close proximity to neighbouring cell populations, they were seen to migrate toward each other and merge (Figure S2.2 C), likely as a result of secreted factors. For this reason, organoids/cell clusters that were in close proximity to another cell population were excluded from the analysis. In some instances, an error occurred with cell tracking, whereby the organoids/clusters selected were lost by cell tracking software (Figure S2.2 D) due to cell migration or plate re-calibration errors and hence not included in the analysis.


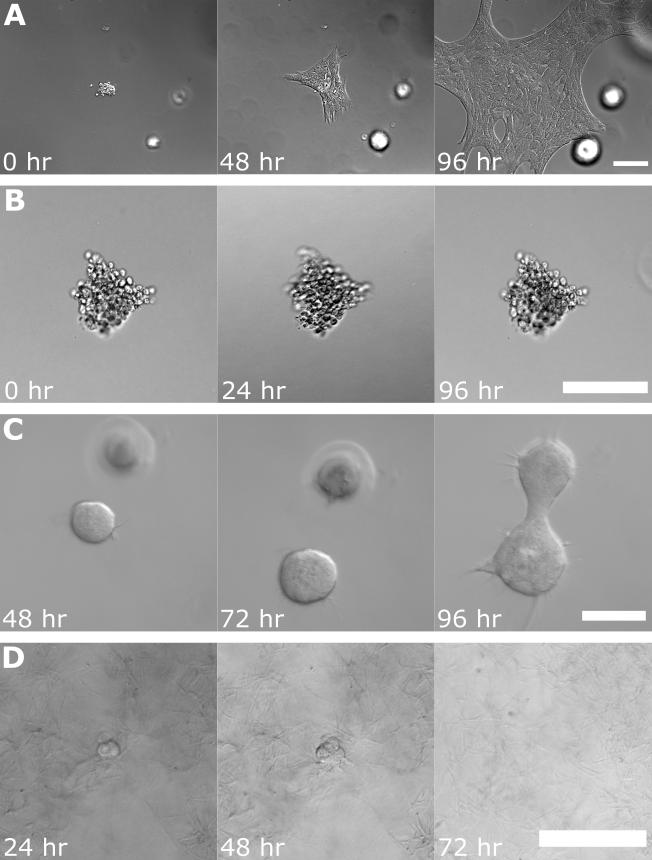


**Figure S2.2.** Technical issues encountered during image analysis. Representative DIC images displaying organoids/cell line clusters that were excluded from the analysis due to technical issues. Organoids/cell clusters were excluded if they adhered to the cell culture plate (**A**), were non-viable (**B**), were plated in close proximity to a neighbouring cell population (**C**) or were lost by cell tracking software (**D**). All scale bars = 100 μm.
